# Clinical Findings in Temporal Lobe Epilepsy Associated With Isolated Amygdala Enlargement

**DOI:** 10.1111/ene.70225

**Published:** 2025-05-27

**Authors:** Annika Kirscht, Johann Philipp Zöllner, Nadine Conradi, Elisabeth Neuhaus, Elke Hattingen, Marcus Belke, Susanne Knake, Laurent Willems, Jennifer Wichert, Andreas Jansen, Felix Rosenow, Adam Strzelczyk

**Affiliations:** ^1^ Department of Neurology and Epilepsy Center Frankfurt Rhine‐Main, Goethe‐University Frankfurt University Hospital Frankfurt Frankfurt am Main Germany; ^2^ Goethe‐University Frankfurt Center for Personalized Translational Epilepsy Research Frankfurt am Main Germany; ^3^ Department of Neuroradiology, Goethe‐University Frankfurt University Hospital Frankfurt Frankfurt am Main Germany; ^4^ Philipps‐University Marburg Department of Neurology, Epilepsy Center Hessen Marburg Germany; ^5^ LOEWE‐Research‐Cluster for Advanced Medical Physics in Imaging and Therapy (ADMIT) TH Mittelhessen University of Applied Sciences Giessen Germany; ^6^ Department of Nuclear Medicine, Goethe‐University Frankfurt University Hospital Frankfurt Frankfurt am Main Germany; ^7^ Philipps‐University Marburg, Department of Psychiatry and Psychotherapy Marburg Germany

**Keywords:** histopathology, neuroimaging, neuropsychology, seizures, temporal lobe

## Abstract

**Background:**

Mesial temporal lobe epilepsy (mTLE) infrequently presents with isolated amygdala enlargement (AE), but its relevance remains ambiguous. We therefore investigated clinical, imaging, and histopathological findings in mTLE‐AE compared to non‐lesional mTLE (mTLE‐NL) patients, and additionally strategies for identifying AE.

**Methods:**

We detected AE by automated volumetry of otherwise unremarkable magnetic resonance images of mTLE patients, compared with a healthy comparator. Autoimmune inflammation as an AE cause was excluded using the Graus criteria. We compared clinical and neuropsychological variables between mTLE‐AE and mTLE‐NL. Secondary assessment of AE was by neuroradiologist visual detection.

**Results:**

Of 63 mTLE patients, 15 had mTLE‐AE. In these, normalized mean volume was 1857.58 mm^3^ (SD = 207.38) for the left, 1973.09 mm^3^ (SD = 214.91) for the right amygdala, 2003.34 mm^3^ (SD = 218.85) for the larger and 1827.34 mm^3^ (SD = 179.85) for the smaller amygdala. Mean volume in the healthy control subjects was 1853.4 mm^3^ for the left (SD = 212.44) and 1895.2 mm^3^ for the right amygdala (SD = 224.29). Clinical parameters including age, sex, epilepsy duration, history of febrile convulsions, drug resistance, neuropsychological performance, surgical outcome, and medications did not differ significantly between mTLE‐AE and mTLE‐NL. Histopathological findings in mTLE‐AE included dysmorphic neurons, potential tumors, and focal cortical dysplasia. Neuroradiologists independently described AE in 37 of 63 mTLE patients.

**Conclusions:**

mTLE‐AE has no specific clinical profile compared to non‐lesional mTLE and features diverse underlying pathologies. Volumetric detection appears more conservative than conventional qualitative visual analysis, but may miss cases of subtle AE. Combining automated volumetry with visual assessment may improve AE detection.

**Abbreviations**
^18^FDG‐PET: 18‐Fluoro‐deoxyglucose positron emission tomography
AE: amygdala enlargement
BDI‐II: Beck Depression Inventory‐II
DCS‐II: diagnostic for cerebral injury II (“Diagnosticum für Cerebralschädigung II”)
EEG: electroencephalogram
FA: flip angle
FCD: focal cortical dysplasia
FLAIR: fluid‐attenuated inversion recovery sequence
HR‐QoL: health‐related quality of life
HS: hippocampal sclerosis
ILAE: International League Against Epilepsy
IQ: intelligence quotient
MRI: magnetic resonance imaging
mTLE: mesial temporal lobe epilepsy
mTLE‐AE: mesial temporal lobe epilepsy with isolated amygdala enlargement
NPT: neuropsychological testing
nTLE‐NL: mesial temporal lobe epilepsy with no evidence of lesion on MRI
PET/CT: positron emission tomography/computed tomography
PR: percentile rank
QoL: quality of life
QOLIE‐31: Quality of Life in Epilepsy Inventory‐31 items
ROCFT: Rey–Osterrieth complex figure test
RWT: Regensburg word fluency test (“Regensburger Wortflüssigkeitstest”)
SD: standard deviation
TAP: alertness test battery (“Testbatterie zur Aufmerksamkeitsprüfung”)
TE: echo time
TI: inversion time
TIV: total intracranial volume
TLE: temporal lobe epilepsy
TR: repetition time
VEM: video electroencephalogram monitoring
VLMT: verbal learning and memorizing test (“Verbaler Lern‐ und Merkfähigkeitstest”)
WMS‐R: Wechsler Memory Scale‐revised

## 1 Introduction

Temporal lobe epilepsy (TLE) is the most prevalent form of focal epilepsy. Mesial TLE (mTLE), its commonest subtype, is most often caused by hippocampal sclerosis (HS) [1, 2]. Isolated amygdala enlargement (AE) has been suggested as another specific finding and possible distinct electroclinical syndrome in mTLE [3]. The amygdala is intertwined with pathophysiological processes in TLE via structure and function, and the extent of amygdala resection correlates with seizure freedom after epilepsy surgery for mTLE [4]. In addition, the amygdala plays an important role in episodic and spatial memory functions as well as emotion regulation, as is evident from postoperative functional deficits as well as seizure semiology in TLE [5, 6, 7].

Several specific causes of AE have been described. Recently, autoimmune mechanisms have attracted considerable attention as a cause of AE [8]. However, autoimmune inflammation is not unequivocally detected in many individuals with epilepsy and isolated AE [9]. Isolated AE has also been associated with other histopathological findings, such as hamartoma and focal dysplasia [10], and epileptogenic tumors, such as oligodendroglioma, ganglioglioma, and astrocytoma [11]. Often, the cause of AE remains undetermined even after extensive diagnostic workup, including histological analysis [12]. Especially in these cases, reactive processes secondary to frequent seizures have been discussed [12]. Therefore, it is impossible to presume a single, common cause for isolated AE in mTLE (mTLE‐AE), and in many cases, AE etiology remains elusive.

Neuropsychological deficits are a common problem in mTLE. Many different cognitive domains can be affected, including memory and executive functions [13]. Both hippocampal and amygdalar dysfunction appear to play a major role in mTLE‐associated memory deficits, and working and episodic memory are particularly affected [14].

The aim of this study was to investigate the presence of a specific clinical and neuropsychological profile in mTLE‐AE that could indicate the distinctiveness of this rare constellation. Although isolated AE is, by definition, primarily detected by amygdala volume, there is no gold standard for distinguishing AE from normal mesiotemporal structure. Thus, we also aimed to compare different detection strategies for AE.

## 2 Methods

### 2.1 Study Design and Population

This study retrospectively included individuals with focal epilepsy and an electroclinical diagnosis of mTLE who underwent inpatient video electroencephalogram (EEG) monitoring (VEM) at the Epilepsy Center Frankfurt Rhine‐Main between March 2016 and January 2022. In all cases, the mTLE diagnosis was confirmed by experienced epileptologists based on the results of the inpatient VEM, according to the International League Against Epilepsy (ILAE) epilepsy classification criteria [15].

We excluded individuals with a priori known epileptogenic lesions (e.g., HS, mesiotemporal atrophy, tumors, vascular malformation) and those with AE due to an a priori known secondary etiology (limbic encephalitis, focal cortical dysplasia [FCD], vascular pathology, and tumors). Autoimmune inflammation was ruled out clinically in all individuals using the Graus criteria [16] and, in 28 individuals, by negative antibody testing.

**FIGURE 1:**
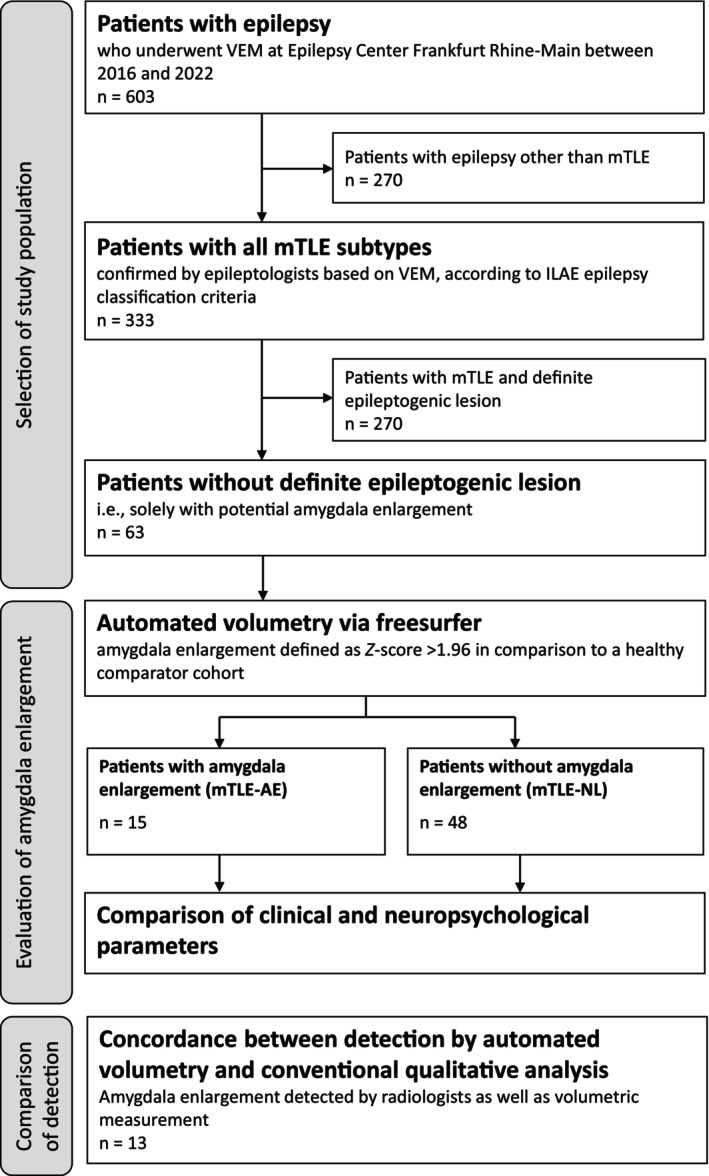
Study design. mTLE, mesial temporal lobe epilepsy; mTLE‐AE, mesial temporal lobe epilepsy with isolated AE on magnetic resonance imaging; mTLE‐NL, non‐lesional mesial temporal lobe epilepsy; VEM, video EEG monitoring.

For the remaining patients without an obvious cause of mTLE, isolated AE was the sole potential morphological correlate. Unequivocal detection of AE is hindered by the fact that its diagnosis relies primarily on amygdala volume, for which there is no established and validated cutoff and thus no diagnostic gold standard. In addition, AE detection on conventional analysis may be biased by individual imaging variables. Thus, we chose (semi‐)automatic volumetry informed by a cohort of healthy individuals as the primary identification method for AE (Figure 1) and by its application divided mTLE patients into (i) those with magnetic resonance imaging (MRI)‐determined isolated AE (mTLE‐AE group) and (ii) those who did not show AE on MRI (non‐lesional group, mTLE‐NL). Further analysis was based on these two groups. Qualitative neuroradiological visual assessment of AE was used as a secondary method to compare automated and conventional AE identification strategies.

Temporal lobe surgery was performed in a subset of mTLE patients according to patients' preferences and the recommendation of an interdisciplinary epilepsy surgery conference.

The study was approved by the ethics committee of the Goethe University Hospital Frankfurt (permit number 367/18).

### 2.2 Imaging

All MR images were acquired using a 3 Tesla MRI scanner (Magnetom Verio/Skyra^fit^; Siemens, Erlangen, Germany) as part of the clinical VEM routine workup. They were qualitatively visually assessed for the presence of epileptogenic and other pathological lesions by board‐certified neuroradiologists experienced in evaluating MRI for presurgical epilepsy workup. Radiologists assessed AE based on its size. Our MRI also included T2‐ and FLAIR sequences in all patients. In postprocessing, only high‐resolution three‐dimensional (3D) T1‐weighted (repetition time [TR] = 2.3 s, echo time [TE] = 0.00232 s, inversion time [TI] = 0.9 s, flip angle [FA] = 8°, voxel‐size = 0.9 mm) and T2‐weighted fluid inversion‐attenuated inversion recovery (FLAIR; TR = 5 s, TE = 0.387 s, TI = 1.8 s, FA = 120°, slice thickness = 0.9 mm) sequences were used for volumetry.

We also used 18‐fluoro‐deoxyglucose positron emission tomography (^18^FDG‐PET) diagnostic scans to evaluate a possible regional cerebral hypometabolism when available. All ^18^FDG‐PET images were acquired and automatically co‐registered on a PET/computed tomography (CT) scanner (Biograph 6; Siemens, Erlangen, Germany) and analyzed by a board‐certified nuclear medicine physician experienced in interpreting PET/CT images for presurgical epilepsy analysis.

### 2.3 Imaging Postprocessing

**FIGURE 2:**
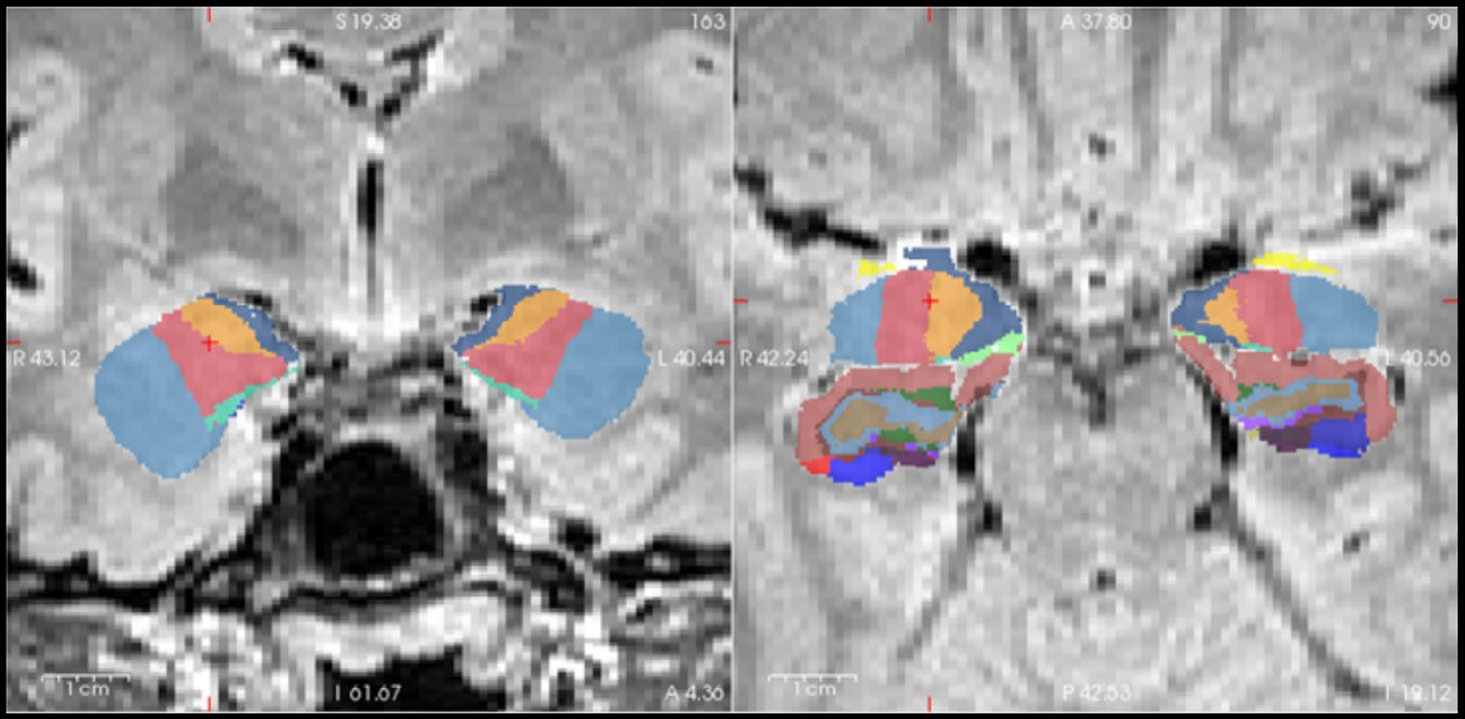
Example of right‐sided amygdala enlargement on automatic segmentation. Exemplary right‐sided amygdala enlargement after multiparametric segmentation. Compared to the healthy control cohort, only the right amygdala was enlarged (*z*‐score = 4.8). The amygdala nuclei are shown in blue/red/orange, and the amygdala‐hippocampal transition zone is shown in light green. The hippocampi are also visualized (coronal and axial T2‐weighted FLAIR sections); FLAIR, fluid‐attenuated inversion recovery.

We determined amygdala volumes using multiparametric volumetry of 3D T1‐ and T2‐FLAIR‐weighted sequences with the automatic brain imaging software FreeSurfer (version 7.4.1) by postprocessing MR images using the standard “recon‐all” pipeline implemented in FreeSurfer [15] with the additional use of the hippocampal subfield segmentation tool [17] (Figure 2). Volumes of subcortical regions were normalized to the brain segmentation volume without ventricles (BrainSegVolNotVent), which was also measured using FreeSurfer.

To correct the volumes for brain size and calculate a *z*‐score, we used an existing cohort of 256 neurologically and psychiatrically healthy subjects who underwent 3D T1‐weighted magnetization prepared‐rapid gradient echo scans (voxel = 1 × 1 × 1 mm, TR = 1900 ms, TE = 2.52 ms, TI = 900 ms, FA = 9°) on a 3 T Trio Scanner (Siemens, Erlangen, Germany) at the Center for Brain Imaging in Marburg, Germany. This healthy control cohort has been previously described [18]. Briefly, the gross amygdala volumes of our subjects were corrected for the total brain size (total intracranial volume, TIV) using a linear regression [18]. These volumetric results were then compared to the TIV‐normalized gross amygdala volumes of the healthy control cohort using *z*‐scores. A *z*‐score ≥ 1.96, reflecting two standard deviations (SDs), was assumed to represent significantly increased amygdala volume compared to healthy controls.

### 2.4 NPT

All included individuals underwent neuropsychological testing (NPT). A standard test battery was used for neuropsychological evaluation, including intelligence, alertness, memory, executive functioning, and spatial and verbal functioning [19]. We selected the most appropriate tests for each tested domain to avoid overlap between similar subtests. Overall intelligence was assessed using the multiple‐choice vocabulary intelligence test (“Mehrfachwahl‐Wortschatz‐Intelligenztest”) and verbal intelligence quotient (IQ) using the Wechsler intelligence test (“Wechsler‐Intelligenztest”). Verbal memory was measured using the verbal learning and memorizing test (VLMT; “Verbaler Lern‐ und Merkfähigkeitstest”), and figural memory using the Diagnostic for Cerebral Injury II (DCS‐II; “Diagnosticum für Cerebralschädigung II”). Auditory and visual memory were assessed using the Wechsler Memory Scale‐Revised (WMS‐R). In this task, the examiner reads aloud sequences of numbers of increasing length, which the subject is then asked to repeat both forwards and backwards. In the visual part, the examiner shows to the subject sequences of increasing length on the block chuck board, which the subject is then asked to repeat both forwards and backwards. Verbal functions were tested using the Regensburg word fluency test (RWT; “Regensburger Wortflüssigkeitstest”), attention using the alertness test battery (TAP; “Testbatterie zur Aufmerksamkeitsprüfung”), and spatial functions using the Rey–Osterrieth Complex Figure Test (ROCFT). Health‐related quality of life (HR‐QoL) was measured using the Quality of Life (QoL) in Epilepsy Inventory‐31 items (QOLIE‐31), and depression was assessed using the Beck Depression Inventory‐II (BDI‐II). Reasons for partly missing test results were necessary to test curtailment due to poor individual compliance (e.g., due to depression or language barriers). All neuropsychological values are given as percentile rank (PR), which means that corrections have already been made for age‐ and education‐associated effects.

### 2.5 Identification of the Dominant Hemisphere

As a part of the presurgical examination, speech lateralization was tested using functional transcranial Doppler sonography [20] and, in the case of uncertain results, the Wada test or functional MRI.

### 2.6 Statistical Analysis

Statistical analyses were performed using SPSS software (version 29; IBM, Armonk, NY, USA). Pearson's product–moment correlation coefficient was used to assess possible correlations between NPT results and AE volumetry. Paired sample *t*‐tests were used to compare parametric NPT variables between individuals mTLE‐AE and mTLE‐NL as well as between individuals with right‐ and left‐sided AE to determine if there were differences in neuropsychological outcomes. Nonparametric variables were compared between these groups using chi‐square tests (Fisher's exact test if the expected cell‐wise frequency was < 5) or Mann–Whitney *U* tests according to data measurement level. *p* values were corrected for multiple testing using the Benjamini and Hochberg method. A two‐sided *p* of < 0.05 was considered statistically significant.

## 3 Results

### 3.1 Study Population and Clinical Characteristics

Of the 333 consecutive individuals with an mTLE diagnosis resulting from VEM at the Epilepsy Center Frankfurt Rhine‐Main between March 2016 and January 2022, 63 had either an isolated AE without a priori evidence of secondary etiology (mTLE‐AE group) or no evidence of epileptogenic lesions (mTLE‐NL group) on MRI. Differentiating these two groups according to the finding of the automatic segmentation and volumetry of the amygdalar nuclei resulted in 15 patients in the mTLE‐AE group and 48 patients in the mTLE‐NL group. The demographic and clinical characteristics of these two groups are shown in Table 1. Among the 63 individuals, the mean age was 43 years (23–72), and 70% were female. Basic clinical variables (sex, age, and drug resistance) did not differ significantly between the mTLE‐AE and mTLE‐NL groups. Although there was a trend toward longer epilepsy duration in the non‐lesional group (mean = 16.5 years, SD = 13.7) than in the mTLE‐AE group (mean = 12.8 years, SD = 11.7), the difference was not significant. Febrile seizures occurred in two individuals in the non‐lesional group and none in the mTLE‐AE group. Drug‐resistant epilepsy was more common in the mTLE‐NL group (81%) than in the mTLE‐AE group (67%). Thirty‐five individuals were tested for lateralization of the language‐dominant hemisphere, of which 30 had left‐sided dominance (86%), four had bilateral dominance (11%), and one had unclear lateralization (3%).

**TABLE 1:** Clinical characteristics of the included patients.

		mTLE‐AE^a^ (*n* = 15)	mTLE‐NL (*n* = 48)	*p*
Sex (male/female), *n*		5/10	14/34	0.757
Age (years), mean (SD)		43.4 (14.9)	43.3 (13.7)	0.981
Epilepsy duration (years), mean (SD)		12.8 (11.7)	16.5 (13.3)	0.170
Febrile seizure (yes/no/undefined)		0/14/1	2/45/1	0.999
Drug‐resistant epilepsy, *n* (%)		10 (66.7)	39 (81.3)	0.270
Number of ASM, median (range)		4 (1–13)	4 (0–16)	0.819
Surgery, *n* (%)		2 (13.3)	7 (14.6)	0.999
Surgery outcome, *n* (%)	Engel IA	0	3 (42.9)	0.500 (Engel IA vs. other outcomes)
	Engel I Other	0	2 (28.6)	Ref.
	Engel II	0	1 (14.3)	Ref.
	Engel III	0	1 (14.3)	Ref.
	Engel IV	2 (100)	0	Ref.

Abbreviations: AE, amygdala enlargement; ASM, antiseizure medication; mTLE‐AE, mesial temporal lobe epilepsy with isolated AE on magnetic resonance imaging; mTLE‐NL, non‐lesional mesial temporal lobe epilepsy; Ref., reference category; SD, standard deviation.

^a^ According to automated volumetry.

### 3.2 MRI and ^18^FDG‐PET

**FIGURE 3:**
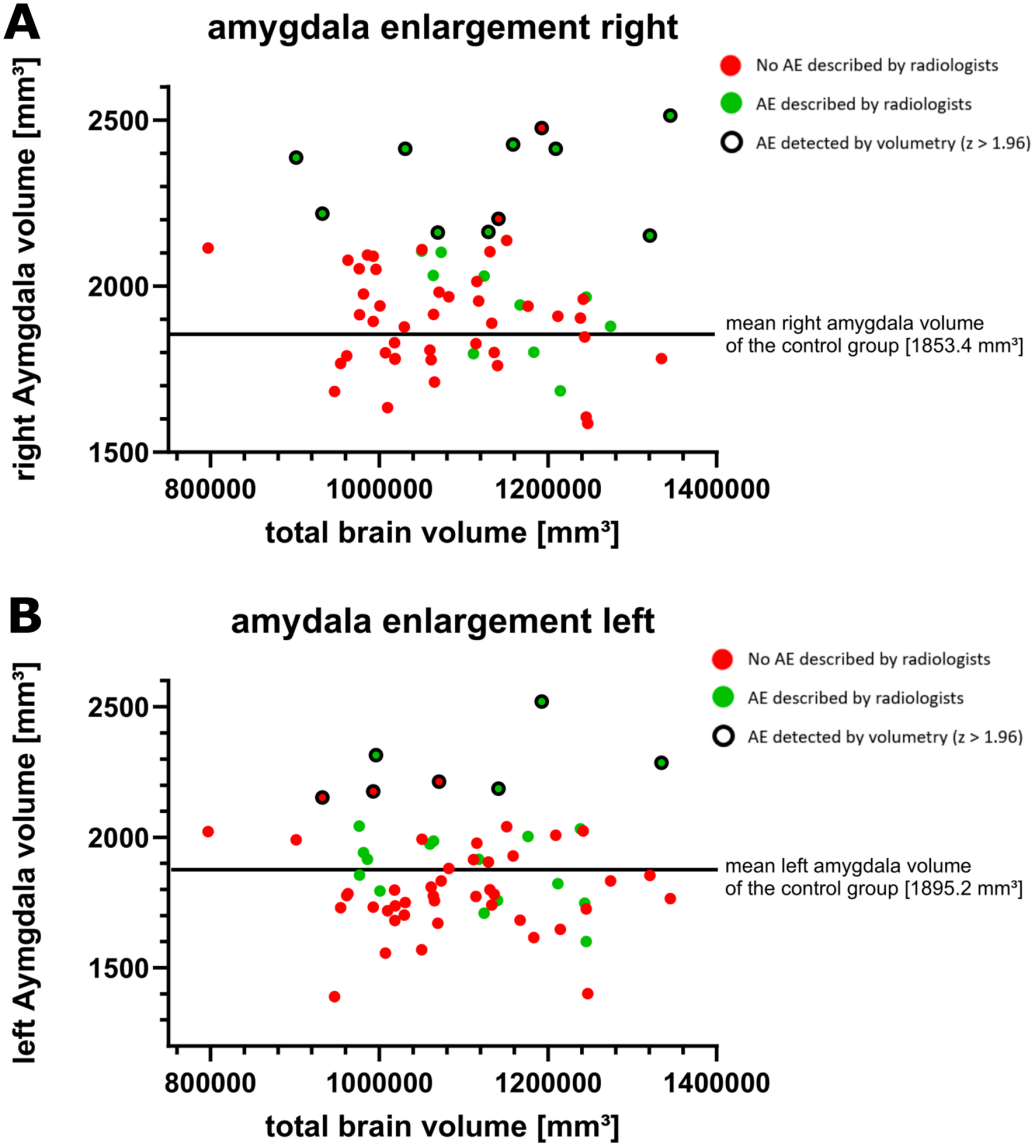
Comparative volumetry results for the mTLE groups, stratified by affected hemisphere. Automated volumetry results of *N* = 63 individuals with mTLE without overt epileptogenic lesions, stratified by hemisphere (right side = A, left side = B). The continuous black line represents the mean amygdala volume in the healthy control cohort. As amygdala volumes are normalized for total brain volume, this line runs horizontally. Circled volumes represent amygdalae determined as enlarged by automated volumetry (corresponding to a *z*‐score > 1.96 above the healthy comparator cohort mean). Additionally, the color of each volumetry result indicates whether conventional qualitative radiological assessment did also diagnose an amygdala enlargement (AE), where red color represents normal amygdala volume according to neuradiologists, and green color represents AE; mTLE, mesial temporal lobe epilepsy.

On automated volumetry, 15 of the 63 individuals showed an AE with a *z*‐score ≥ 1.96 above the healthy control cohort; an example is provided in Figure 2. Eight individuals demonstrated right‐sided AE, four showed left‐sided AE, and three showed bilateral AE. The mean volume was 1857.58 mm^3^ (1389.25–2519.99; SD = 207.38) for the left amygdala and 1973.09 mm^3^ (1586.32–2512.92; SD = 214.91) for the right amygdala. The mean volume was 2003.34 mm^3^ (1586.32–2519.99; SD = 218.85) for the larger amygdala and 1827.34 mm^3^ (1389.25–2476.09; SD = 179.85) for the smaller amygdala. A visual comparison to the healthy control cohort is provided in Figure 3.

In contrast, 37 of the 63 individuals showed AE according to standard neuroradiological qualitative visual assessment: Right‐sided AE was described in 18 (48.6%), left‐sided AE in 18 (48.6%), and bilateral AE in one (2.7%).

**FIGURE 4:**
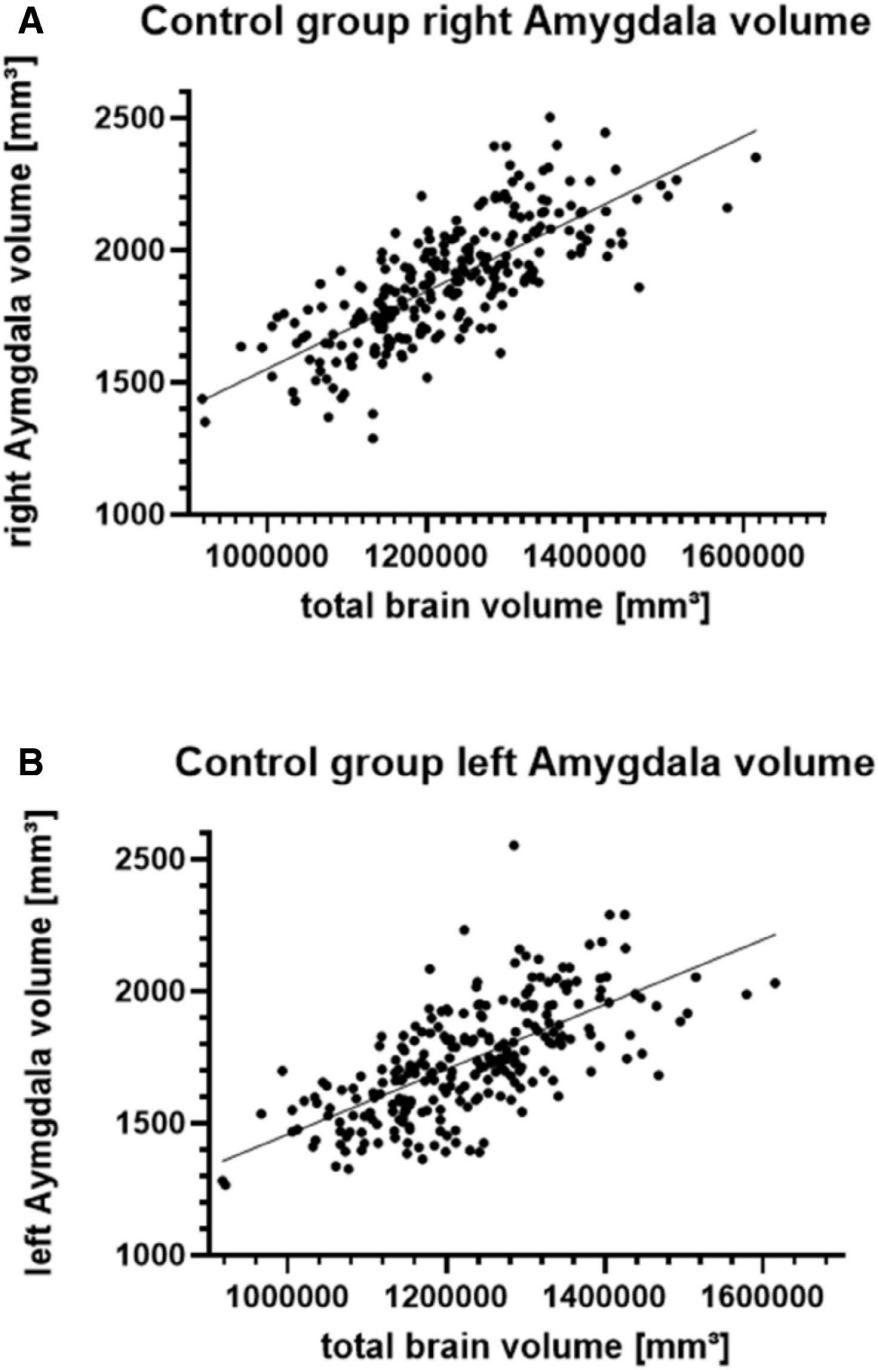
Volumetry results for the healthy comparator cohort, stratified by hemisphere. Normalized volumetry results of healthy individuals, as determined by automatic volumetry (right side = A, left side = B). The continuous black line represents the mean amygdala volume. Note the corresponding increase of amygdala volume with increasing total brain volume.

Figure 4 shows all amygdala volumes relative to the total brain volume in the healthy control cohort. As expected, amygdala volume increases with brain size. In the healthy control subjects, the mean volume was 1853.4 mm^3^ for the left amygdala (1266–2552; SD = 212.44) and 1895.2 mm^3^ for the right amygdala (1287–2503; SD = 224.29).

The qualitative visual assessment and automated volumetry results matched in 59% of cases (37/63). In 13 cases, AE was detected by the neuroradiologist and by automated volumetry. In contrast, in two cases, AE was detected by automated volumetry but not initially detected by qualitative visual assessment. Conversely, in 24 cases, AE was described by the radiologists but was not identified by automated volumetry. Notably, AE was neither detected by automated volumetry nor by qualitative visual assessment in 24 individuals. We also correlated the hypometabolism in ^18^FDG‐PET images with the automatically measured sizes of the amygdala. However, there was no significant correlation between hypometabolism in ^18^FDG‐PET and an enlarged amygdala.

### 3.3 Neuropathological Findings

Of the 63 included individuals, nine underwent epilepsy surgery. In three cases, this was temporal pole resection with amygdalohippocampectomy, and in six cases, anterior temporal lobectomy (including amygdalohippocampectomy) was performed. Of all patients who underwent surgery, two had an AE according to automated volumetry (see below) and showed differing histological findings (one had a potential tumor, and one demonstrated dysmorphic neurons within the amygdala). Despite the amygdala being removed, both individuals did not achieve seizure freedom (Engel IV). The other seven individuals also showed varying findings; three had nonspecific histological findings, one had dysmorphic neurons, and three had definite FCD type II of the temporal neocortex eventually detected. Five of these seven individuals achieved seizure freedom (Engel I), one achieved Engel II, and one achieved Engel III.

### 3.4 Neuropsychological Examination

HR‐QoL (as tested using the QOLIE‐31 questionnaire) did not differ significantly between the mTLE‐AE (PR = 44) and mTLE‐NL (PR = 43) groups. However, individuals with left‐sided AE reported a slightly better HR‐QoL (PR = 46) than those without AE (PR = 40; *p* = 0.041). Nevertheless, both test results were below the mean of 50 and, therefore, below average. In the depression testing, the mean BDI‐II score was 14.2 (SD = 12.0) in the mTLE‐AE group; in the mTLE‐NL group, it was 15.93 (SD = 8.8). In addition, the mean IQ was 100.31 (SD = 11.9) in the mTLE‐AE group and 102.06 (SD = 12.5) in the mTLE‐NL group. Individuals with mTLE‐AE performed slightly better in the ROCFT delayed free recall subtest, with a mean SD of −0.5 in the mTLE‐AE group and −1.0 in the non‐lesional mTLE group.

### 3.5 Differences Between Individuals With and Without AE

Most neuropsychological categories demonstrated slightly better performance in the mTLE‐AE group than in the non‐lesional mTLE group, but without reaching significance (Table 2). The mTLE‐AE group performed slightly better than the non‐lesional mTLE group in the auditory and visual memory tests (WMS‐R). The mean PR was 45 in the mTLE‐AE group and 37 in the mTLE‐NL group when recalling numbers in the auditory memory test, a difference of 8. Verbal memory capacity was the same in both groups, with a PR of 45 in the mTLE‐AE group and 44 in the non‐lesional mTLE group. Only in the subtest “delayed free recall” did the mTLE‐AE group (PR = 29) perform worse than the non‐lesional mTLE group (PR = 34). Figure memory performance was better in the mTLE‐AE group (PR = 40) than in the non‐lesional mTLE group (PR = 34).

**TABLE 2:** Neuropsychological test results in patients with (*n* = 15) and without (*n* = 48) AE.

Neuropsychological test	Group	Mean^a^	*p* ^b^	Corrected *p* ^c^
Divided attention (TAP: missed sounds or squares in total)	mTLE‐AE	29.82	0.478	> 0.999
	mTLE‐NL	37.22		
Memory span (WMS‐R: auditory number span forward)	mTLE‐AE	45.42	0.374	> 0.999
	mTLE‐NL	36.97		
Memory span (WMS‐R: auditory number span backwards)	mTLE‐AE	38.75	0.819	> 0.999
	mTLE‐NL	36.46		
Memory span (WMS‐R: visual block span forward)	mTLE‐AE	50.17	0.222	> 0.999
	mTLE‐NL	37.20		
Memory span (WMS‐R: visual block span backwards)	mTLE‐AE	46.42	0.145	> 0.999
	mTLE‐NL	32.00		
Verbal memory (VLMT5: capacity)	mTLE‐AE	44.58	0.979	> 0.999
	mTLE‐NL	44.35		
Verbal memory (VLMT5‐7: delayed recall)	mTLE‐AE	28.92	0.608	> 0.999
	mTLE‐NL	33.56		
Figural memory (DCS‐II: learning performance)	mTLE‐AE	39.78	0.625	> 0.999
	mTLE‐NL	33.81		
Executive functioning word fluency (RWT: form lexically simple)	mTLE‐AE	38.63	0.931	> 0.999
	mTLE‐NL	39.61		
Executive functioning word fluency (RWT: semantically)	mTLE‐AE	51.82	0.306	> 0.999
	mTLE‐NL	39.74		
Reaction flexibility (TAP: reaction times)	mTLE‐AE	47.70	0.451	> 0.999
	mTLE‐NL	39.81		
Reaction flexibility (TAP: error reactions)	mTLE‐AE	62.10	0.467	> 0.999
	mTLE‐NL	53.94		

Abbreviations: AE, amygdalar enlargement; DCS‐II, Diagnostic for Cerebral Injury‐II; mTLE‐AE, mesial temporal lobe epilepsy with isolated AE on magnetic resonance imaging; mTLE‐NL, non‐lesional mesial temporal lobe epilepsy; RWT, Regensburg word fluency test; TAP, divided attention test battery; VLMT5, verbal learning and memory test after 5 learning cycles; VLMT5‐7, verbal learning and memory test difference between the learning performance after the 5th learning cycle (VLMT5) and the VLMT7 (correct reproduction after a time delay); WMS‐R, Wechsler Memory Scale‐Revised.

^a^ All values are T‐scores unless otherwise noted.

^b^ *t*‐test.

^c^ Corrected for multiple testing according to Benjamini and Hochberg's method.

Executive functioning did not differ significantly between the mTLE‐AE and mTLE‐NL groups. However, responsiveness was slightly better in the mTLE‐AE group. Reaction times were faster in the mTLE‐AE group (PR = 48) than in the non‐lesional mTLE group (PR = 40). In addition, on average, there were fewer false reactions in the mTLE‐AE group (PR = 62) than in the non‐lesional mTLE group (PR = 54). However, these differences in PR were not significant. We also compared the neuropsychological results of the mTLE‐AE group with the mTLE‐NL group, with groups alternatively determined by conventional radiological analysis. Again, the mTLE‐AE and mTLE‐NL groups did not differ significantly. However, notably, the mTLE‐NL group tended to perform worse in the tests (Table 3).

**TABLE 3:** Neuropsychological test results in patients with (*n* = 37) and without (*n* = 26) radiology‐defined AE.

Neuropsychological test	Group	Mean^a^	*p* ^b^	Corrected *p* ^c^
Divided attention (TAP: missed sounds or squares in total)	mTLE‐AE	39.82	0.173	> 0.999
	mTLE‐NL	26.93		
Memory span (WMS‐R: auditory number span forward)	mTLE‐AE	44.20	0.115	> 0.999
	mTLE‐NL	30.18		
Memory span (WMS‐R: auditory number span backwards)	mTLE‐AE	41.93	0.133	> 0.999
	mTLE‐NL	28.41		
Memory span (WMS‐R: visual block span forward)	mTLE‐AE	42.67	0.538	> 0.999
	mTLE‐NL	36.71		
Memory span (WMS‐R: visual block span backwards)	mTLE‐AE	37.07	0.697	> 0.999
	mTLE‐NL	33.53		
Verbal memory (VLMT5: Capacity)	mTLE‐AE	49.14	0.097	> 0.999
	mTLE‐NL	36.35		
Verbal memory (VLMT5‐7: delayed recall)	mTLE‐AE	31.97	0.900	> 0.999
	mTLE‐NL	33.00		
Figural memory (DCS‐II: learning performance)	mTLE‐AE	37.68	0.573	> 0.999
	mTLE‐NL	31.57		
Executive functioning word fluency (RWT: form lexically simple)	mTLE‐AE	41.88	0.499	> 0.999
	mTLE‐NL	35.47		
Executive functioning word fluency (RWT: semantically)	mTLE‐AE	44.69	0.662	> 0.999
	mTLE‐NL	40.00		
Reaction flexibility (TAP: reaction times)	mTLE‐AE	39.67	0.501	> 0.999
	mTLE‐NL	45.71		
Reaction flexibility (TAP: error reactions)	mTLE‐AE	59.11	0.364	> 0.999
	mTLE‐NL	49.79		

Abbreviations: AE, amygdala enlargement; DCS‐II, Diagnostic for Cerebral Injury‐II; mTLE‐AE, mesial temporal lobe epilepsy with isolated AE on magnetic resonance imaging; mTLE‐NL, non‐lesional mesial temporal lobe epilepsy; RWT, Regensburg Word Fluency Test; TAP, divided attention test battery; VLMT5‐7, verbal learning and memory test difference between the learning performance after the 5th learning cycle (VLMT5) and the VLMT7 (correct reproduction after a time delay); WMS‐R, Wechsler Memory Scale‐Revised, VLMT5, verbal learning and memory test after 5 learning cycles.

^a^ All values are T‐scores unless otherwise noted.

^b^ *t*‐test.

^c^ Corrected for multiple testing according to Benjamini and Hochberg's method.

## 4 Discussion

Mesial temporal lobe epilepsy associated with an isolated AE (mTLE‐AE) is a comparatively rare constellation in the presurgical epilepsy diagnostic setting. Little is currently known about the specific neuropsychological profile in individuals with mTLE‐AE. In addition, a specific cutoff for an enlarged amygdala has not yet been defined. Therefore, our study aimed to describe the clinical and neuropsychological characteristics as well as histopathological findings of patients with mTLE‐AE and identify optimal detection/distinction strategies.

### 4.1 General Clinical Characteristics

Overall, our study demonstrated that clinical profiles of individuals with mTLE‐AE are similar to those with non‐lesional mTLE (Table 1). In both the mTLE‐AE and mTLE‐NL groups, the time to epilepsy diagnosis was considerably longer than usually described for MRI‐negative extratemporal focal epilepsy [21]. The combination of seizure semiology and the lack of salient morphological features, which increase the difficulty of diagnosis, likely explains the trend toward a relatively longer diagnostic delay in the non‐lesional mTLE cohort. Interestingly, however, this delay was not significantly different from the mTLE‐AE cohort, suggesting that isolated AE as a morphological correlate of epilepsy does not expedite diagnosis over non‐lesional (“MRI‐negative”) epilepsy, likely due to its relatively recent description. The prevalence of febrile seizures also did not differ significantly between groups, suggesting that, unlike in HS‐related mTLE, febrile seizures are not a specifically mTLE‐AE‐associated condition. Excellent versus non‐excellent surgical outcomes did not differ significantly between AE groups. However, our sample size was limited, which precludes further interpretation.

### 4.2 Comparison of AE Assessment Methods

Since there is no universally accepted diagnostic criterion for AE, its identification method is paramount to its clinical characterization. In our analysis, no single method emerged as clearly superior. Visual assessment may help to recognize an abnormal amygdala more precisely by including, for example, signal intensity abnormalities. Our results suggest that AE is more likely to be falsely seen qualitatively in individuals with generally larger total brain volumes (Figure 3). Therefore, a combination of automated volumetric analysis and visual assessment of signal intensity alterations seems to be a suitable method for detecting AE.

### 4.3 Memory Deficits

Individuals with TLE often suffer from neuropsychological deficits, especially in memory, as relevant structures are located within the epileptogenic networks [22]. As HS is the most common mTLE subtype, most studies on TLE and neuropsychological performance examined changes in the hippocampus and its effect on memory or executive functions. Griffith et al. [23] described the role of the left hippocampus in delayed verbal memory. McDonald et al. [24] also showed that the volume of the left hippocampus significantly influenced verbal memory function. To our knowledge, the specific neuropsychological deficit profile in individuals with mTLE‐AE and non‐lesional mTLE has not yet been examined.

Our study found a general nonsignificant trend toward better neuropsychological performance in those with mTLE‐AE than in those with mTLE‐NL. However, neuropsychological test scores were generally similar between these two groups. We found that visual memory was significantly better in those with mTLE‐AE. However, there was no significant correlation between the (volumetric) extent of mTLE‐AE and memory deficits within the mTLE‐AE group. Previous research has indicated a relationship between amygdala volume and visual memory in age‐related memory decline [25]. That this relationship does not hold in our epilepsy cohort may be due to the differing mechanisms in epilepsy and spontaneous brain aging and potential postictal volumetric changes in mesial temporal structures.

Typically, in right‐handed individuals, the language‐dominant side is on the left, and the nonlanguage‐dominant side is on the right [26]. All individuals with mTLE‐AE in our cohort were language dominant in the left hemisphere. Therefore, it might be expected that those with left‐sided AE would perform worse on the verbal memory tests in our cohort, like in HS‐related mTLE. However, we did not find a significant difference in verbal memory test scores between right‐ and left‐sided mTLE‐AE, suggesting that AE alone does not confer similar structure–function associations as HC.

### 4.4 Executive Functions and Alertness

Executive functions are usually located in the frontal lobe, but corresponding changes have increasingly been described in TLE [27]. Alertness is also associated with frontal structures. In frontal brain damage, reaction times are often delayed [28]. mTLE, especially in HS, not only leads to memory deficits typical of the temporal lobe but is also associated with extratemporal deficits such as attention and executive function [13]. However, individuals with mTLE‐AE do not appear to have major deficits in these areas, possibly due to a generally milder phenotype or relatively spared functional connections to the frontal lobe [29].

### 4.5 QoL

Epilepsy commonly reduces affected individuals' QoL [30]. The average T‐score for the QOLIE‐31, reflecting QoL, in the AE group was 44.0, significantly lower than the average T‐score of 54.5 for individuals with epilepsy in Germany [31]. There was no significant difference in QoL between individuals with mTLE‐AE and mTLE‐NL in our cohort. Pauli et al. showed that QoL in individuals with HS‐related mTLE averages 40.8 [32]. Therefore, the QoL of individuals with mTLE‐AE seems comparable to that of individuals with HS‐related mTLE.

### 4.6 Limitations and Outlook

This was a cross sectional study. A longitudinal study would be desirable to better understand the subtype of mTLE‐AE. In particular, a longitudinal analysis of the effect of seizure frequency on amygdala size and neuropsychological functions in this cohort would be of great interest. Regarding comparison between automated and conventional visual qualitative assessment, neuroradiologists used all available sequences. Although patients with overt epileptogenic lesions in the amygdala were excluded from our analysis, we cannot rule out that subtle signal intensity changes in the amygdala on T2‐weighted or FLAIR imaging may have influenced size assessment during visual quantitative assessment and thus introduced a bias compared to automated volumetry. In addition, it should be mentioned that FreeSurfer has some methodological weaknesses regarding the segmentation of the amygdala as described by Sadil et al. [33]

## 5 Conclusions

Our study demonstrated that individuals with mTLE associated exclusively with AE had similar neuropsychological deficits to those with non‐lesional TLE on MRI. QoL in mTLE‐AE was similar to that in HS‐associated mTLE. mTLE‐AE is likely not caused by a single factor but by different pathologies. Volumetric assessment appears to be better than conventional qualitative radiological assessment in ruling out falsely assumed mTLE‐AE, but slight AE or in situ amygdala lesions may be missed.

## Author Contributions

**Annika Kirscht:** methodology, investigation, writing – original draft, formal analysis. **Johann Philipp Zöllner:** conceptualization, methodology, investigation, writing – review and editing. **Nadine Conradi:** data curation, writing – review and editing. **Elisabeth Neuhaus:** data curation, writing – review and editing. **Elke Hattingen:** writing – review and editing. **Marcus Belke:** resources, writing – review and editing. **Susanne Knake:** writing – review and editing. **Laurent Willems:** visualization, writing – review and editing. **Jennifer Wichert:** data curation, writing – review and editing. **Andreas Jansen:** data curation, writing – review and editing. **Felix Rosenow:** supervision, writing – review and editing. **Adam Strzelczyk:** supervision, writing – review and editing.

## Ethics Statement

We confirm that we have read the Journal's position on issues involved in ethical publication and affirm that this report is consistent with those guidelines.

## Conflicts of Interest

J.P.Z. received speaker's honoraria from Danone and Jazz Pharmaceuticals outside the submitted work. S.K. received speakers' honoraria from Bial, Desitin Arzneimittel, Eisai, Jazz Pharma, Merck Serono, and UCB. F.R. reports honoraria for scientific advice and as speaker from Angelini Pharma, Eisai Pharma, Jazz Pharmaceuticals, Stoke Therapeutics, Takeda, and UCB Pharma. Research support: European Union (EU‐FP7), German Research Foundation (DFG), Federal State of Hesse, Germany, Detlev Wrobel Fonds for Epilepsy Research, Dr. Reiss‐Stiftung, Dr. Senckenbergische‐Stiftung, Kassel‐Stiftung, Ernst Max von Grunelius‐Stiftung, Chaja‐Stiftung, Desitin Arzneimittel, Dr. Schär Deutschland GmbH, Nutricia Milupa GmbH, and Vitaflo Deutschland GmbH. A. Strzelczyk received personal fees and grants from Angelini Pharma, Biocodex, Desitin Arzneimittel, Eisai, Longboard, Jazz Pharmaceuticals, Neuraxpharm, Stoke Therapeutics, Takeda, UCB Pharma, and UNEEG medical. None of the other authors have any conflicts of interest to disclose.

## Data Availability

The data that support the findings of this study are available from the corresponding author upon reasonable request.
